# The Story of the Insane from Year to Year

**Published:** 1894-06-30

**Authors:** 


### THE INSTITUTIONAL WORKSHOP.

### THE STORY OF THE INSANE FROM YEAR TO YEAR.

### MARYLAND HOSPITAL FOR THE INSANE.

This hospital or asylum, as we should call it on this side of the Atlantic, is situated near Catonsville in Baltimore County. At the beginning of the year it contained 427 patients, and at the close of the year there were 461, which shows that the United States, like the old country, is adding to her insane population. The admissions reached 122, the recoveries 25, and the deaths 44. In addition to the recoveries 16 were discharged improved. Among the admissions were 13 Germans, two Irishmen, two Englishmen, and one Welshman. In several Scotch asylums the walls of the airing courts have been pulled down, a proceeding which we hope to see carried out in England, and at Maryland Dr. Rohe tells us that the high fence around the women's airing ground was blown down by a storm, and he obtained the consent of the governing body to remove it entirely. No attempts have been made by the patients to go beyond the boundary, whereas "formerly climbing the high fence and attempts to escape were not infrequent." It would seem, too, that the open door system is in vogue, for we read that "in some of the wards the patients go and come at pleasure," and for mechanical restraint, it is quite unknown. Dr. Rohe is of opinion that removal of the ovaries is curative or alleviative in some forms of insanity, but he is inclined to limit operative interference to those cases where sufficient disease exists to require treatment on its own account, and he believes the mental disturbance is an additional reason for early and effective interference. Details of 22 cases are given, and it seems that three cases of profound melancholia recovered sufficiently to be discharged. One case of hystero-epilepsy of eight years' duration was completely cured. In four cases of puerperal insanity, two of them being of over five years' standing, three recoveries followed the operation and the fourth case was greatly improved. These results are most encouraging, and well deserve careful study by our alienist physicians. The report is a very interesting one, and contains papers by the other medical officers, of whom there are four.

### HOLLOWAY SANATORIUM, VIRGINIA WATER.

The table on page 39 of the report of this asylum tells us that the numbers increased from 334 to 361; and it is now the largest hospital of its kind in England. The admissions reached the respectable figure of 202; the recoveries were 83, and the deaths 28. The percentage of recoveries on admissions was 50·3, and the deaths work out at 8 per cent. on the average number resident. Hereditary influence accounts for 54 of the admissions, and drink for 12; while mental anxiety is said to have been the cause in 55 cases. There is a useful rule in force in this asylum, which requires that one-fourth of the patients shall be maintained at 25s. a week or under, one-fourth at rates between 25s. and 42s., and one-half at a rate which may be agreed upon; and Dr. Philipps says this rule has been carried out in a liberal spirit. A table is given showing the number of patients who received charitable assistance during the year, and it is stated that this assistance amounted to £4,407. The paragraph on official visitation is worth quoting in full, not because the ideas expressed are new, but because such ideas deserve greater publicity than they are likely to meet with in an asylum report. "It has always seemed to me," says Dr. Philipps, "that the present system of lunacy inspection is as wasteful and absurd as it is altogether inadequate. The Lunacy Commission is much undermanned, and yet its work often overlaps that of the Chancery visitors. Three medical Commissioners are supposed to be equal to the efficient supervision of 89,822 insane patients. Two medical visitors inspect a few hundred Chancery patients, all of whom are also visited by the Lunacy Commissioners. Many of the cases inspected by the Commissioners are recent or acute, and possibly doubtful; the Chancery visitors only deal with cases which are chronic, and therefore presumably incurable. Surely changes are called for, which might well take the shape of amalgamation of Government inspecting bodies, a transference of the supervision of all pauper lunatics to the Poor Law division of the Local Government Board, and the appointment of district inspectors. Then it would be practicable for every new case admitted into any institution for the insane to receive prompt official skilled inspection, a condition of things which cannot be guaranteed at present." We are pleased to note that Dr. Philipps continues the lectures to his nursing staff, and that nine nurses and ten attendants have passed the examinations of the Medico-Psychological Association. "The committee desire to express their appreciation of the continued zeal and ability displayed by the Medical Superintendent." The average weekly cost per head is £2 5s.

### EASTERN ASYLUM FOR IDIOTS AT COLCHESTER.

This is a "charitable institution" receiving idiot children from the counties of Essex, Suffolk, Norfolk, and Cambridge. The patients are elected for a period of five years, but this period may be extended from time to time if the necessity of any case seems to require it. Paying cases are also received. While recognising the good which is being done by this asylum and by kindred institutions, we are satisfied that the care of these idiots and imbeciles should be the duty of the State, and should not be left to the donations of the charitable. Last year a sum of £3,700 was spent on these cases in this asylum alone, and there can hardly be a doubt that the most of it should have been paid out of the rates; but so long as people can be found to do the work of the State, so long will the State neglect its duty. Had there been idiot asylums in Middlesex, Warwick, Northampton, Surrey, or Hampshire, not one of these counties would have made provision for its idiot children. We are also of opinion that no asylum at the present day should be under lay superintendence, no matter how well it may seem to answer in individual instances. Where patients of any kind are massed by the hundred, a resident medical officer is essential, and it is evident that this medical officer should be at the head of the establishment. From Mr. Turner's report (which is very clear and well arranged) we learn that the numbers increased during the year from 213 to 233. There were 35 admissions and only 10 deaths. To illustrate the apparent hopelessness of the cases sent to these idiot asylums we may quote Mr. Turner's remarks on the admissions of the year. "Their condition may be inferred when I say that 5 could not walk, 14 were more or less uncleanly in their habits, 3 could not speak, 22 could speak imperfectly, and 10 could not feed themselves." There would seem to be plenty of entertainments provided for the patients; and there is a probability of a seaside house being added to the resources of the asylum. Many minor improvements have been carried out during the year. We are pleased to note that Nurse Barkway was presented with a gold watch and chain on the completion of thirty years' service. There can be no doubt that she deserved it. The managers say that "the best thanks of the board are due to Mr. and Mrs. Turner for their continued zeal and energy in carrying on the work of the asylum." The Commissioners' report is not given, and most of the tables usually printed with asylum reports are absent. The weekly rate per head is 11s. 2¼d., by no means an extravagant rate when it is remembered that the average number of patients resident is only 219.

